# Modification of Spanish Mackerel (*Scomberomorus niphonius*) Surimi Gels by Three Anionic Polysaccharides

**DOI:** 10.3390/foods14152671

**Published:** 2025-07-29

**Authors:** Zhu-Jun Zhang, Fan-Yu Kong, Lin-Da Zhang, Miao-Miao Luo, Yin-Yin Lv, Ce Wang, Bin Lai, Li-Chao Zhang, Jia-Nan Yan, Hai-Tao Wu

**Affiliations:** 1 SKL of Marine Food Processing & Safety Control, National Engineering Research Center of Seafood, Collaborative Innovation Center of Seafood Deep Processing, School of Food Science and Technology, Dalian Polytechnic University, No. 1, Qinggongyuan, Dalian 116034, China; layz02007@163.com (Z.-J.Z.); kfy9952025@163.com (F.-Y.K.); zld13194226841@163.com (L.-D.Z.); duola0401@163.com (M.-M.L.); 13931053789@163.com (Y.-Y.L.); wangceyx@163.com (C.W.); laibin@dlpu.edu.cn (B.L.); 2 Institutes of Biomedical Sciences, Shanxi University, Taiyuan 030006, China; zlc@sxu.edu.cn

**Keywords:** Spanish mackerel surimi, polysaccharides, gel properties, thermal stability, microstructures

## Abstract

This study investigated the gel performance of Spanish mackerel surimi gels (SMSGs) modified by three anionic polysaccharides: κ-carrageenan (KC), ι-carrageenan (IC), and gellan gum (GG). By incorporating polysaccharides, SMSGs showed a 24.9–103.4% improvement in gel and textural properties, in which KC and IC had more improvement effects than GG. Moreover, polysaccharides led to a 10.7–13.1% increment in WHC, a shortened water migration from 61.34 to 52.43–55.93 ms in *T*_22_, and enhanced thermal stability of SMSGs. The content of α-helix in SMSGs reduced markedly accompanied by a concurrent enhancement of β-sheet and β-turn by adding polysaccharides, where β-sheet and β-turn are positively correlated with hardness being favorable for gelling. The microstructure of SMSGs/polysaccharides showed a homogeneous network mainly due to hydrophobic interactions and disulfide bonds in SMSG-based gels. This study will demonstrate the effectiveness of KC, IC, and GG in improving the texture and functionality as well as expanding the application of surimi products.

## 1. Introduction

Surimi is a high-grade myofibrillar protein concentrate that is an intermediate product produced after deboning, chopping, and washing processing to abolish sarcoplasmic proteins, lipids, blood, etc. [1]. Surimi-based products such as fish balls, crabsticks, paupiettes, and fish cakes have become increasingly consumed foods owing to their unique texture, pleasant flavor, and high nutritional value [2]. Spanish mackerel (*Scomberomorus niphonius*), as a vital marine economic fish exhibiting perceived health benefits, is widely distributed in the East China Sea [3,4], and it has been commonly used to produce surimi products due to its tender flesh as well as ease of deboning. In total, the production of Spanish mackerel in China exceeded 367,000 tons in 2023. Indeed, surimi products made from pure Spanish mackerel display weak gel-forming ability with low acceptability [4], and the mechanism behind this is still not clear. Collectively, aquatic animals are poor in salt-soluble proteins and rich in endogenous proteases, which could disrupt the reticulation structure formed by actinomyosin molecules, thus leading to poor gel behavior [5,6]. However, relevant studies are only focused on the modification of certain processing steps and the utilization of physical technologies to enhance the gel properties of SMSGs, including modifying the washing process [4] and the use of high pressure [7]. Thus, it is essential to seek other effective methods to modify the gelling properties of SMSGs.

Nowadays, exogenous additives are widely applied to surimi gels during processing to improve their qualities, including enzymes [8], proteins [9], polyphenols [10], and polysaccharides [1]. Among them, most studies have concentrated on the addition of polysaccharides to surimi systems to tailor their gel properties to a preferred texture, which could efficiently expand their processing and utilization by reducing production costs [11,12]. During the heating process, introducing polysaccharides at relatively low concentrations could affect the hydrogen bonding, hydrophobic forces, and disulfide bonds of proteins present in surimi gels, thus forming a more stable three-dimensional network [13]. Currently, polysaccharides with different charge characteristics have been widely used in modifying surimi gels, such as κ-carrageenan (KC) for golden pompano surimi [14], ι-carrageenan (IC) for Alaska pollock surimi [15], gellan gum (GG) for bigeye snapper surimi [16], chitosan for croaker surimi [17], and konjac glucomannan for Alaska pollock surimi [2]. These polysaccharides might have distinct effects on the gelation of surimi proteins because of the significant differences in their molecular weights, charge densities, and monosaccharide compositions [18].To some extent, polysaccharides with lower molecular weights interact more readily with proteins than their polymeric counterparts due to their less steric hindrance [19]. Moreover, higher-charge-density polysaccharides could more effectively lead to stronger gel formation [18]. In particular, three anionic polysaccharides, KC, IC, and GG, are considered representative polysaccharides applied in reconstituted foods due to their relatively high negative charge and low molecular weights [16,20]. In detail, KC and IC are noted to enhance the WHC as well as the rheological properties of surimi products and are commonly utilized as gelling agents [15]. GG exhibits great processability and suitable rheological properties, and is able to improve surimi properties [16,21]. Moreover, our preliminary study has investigated the influence of polysaccharides with different electrical characteristics on the physicochemical properties of SMSGs, where the KC-, IC-, and GG- treated groups show superior gel performance compared to the other groups. Therefore, we hypothesized that adding these anionic polysaccharides would be a feasible method to increase the gel properties and utilization value of SMSGs, which should be further explored.

The purpose of this work was to analyze the influence of three anionic polysaccharides (KC, IC, and GG) on the gel behavior, structural properties, and potential mechanisms of SMSGs. These results might provide scientific support for commercial application of polysaccharides in SMSGs for developing highly nutritional surimi.

## 2. Materials and Methods

### 2.1. Materials and Chemicals

The deceased Spanish mackerel (1.2–1.5 kg) was purchased from Qianhe seafood market (Dalian, China). κ-carrageenan and IC were provided by Aladdin Co., Ltd. (Shanghai, China). Gellan gum was provided by Sangon Biotech Co., Ltd. (Shanghai, China). All applied chemicals and reagents were of analytical quality.

### 2.2. Preparation of SMSGs

Spanish mackerel was frozen immediately after being caught. Then, the frozen Spanish mackerel was subsequently placed at 25 °C for about 40 min to ensure it was in a semi-frozen state. The heads of the fresh fish were removed by professionally trained personnel and then transported to the laboratory on ice within 30 min. Next, the skin, guts, and red muscles of fish were taken out manually and cut into small cubes. After that, the cubes were rinsed twice using 1:5 (*v/w*) distilled water (4 °C) for 10 min and washed once with 0.3% (*w/w*) NaCl (4 °C) for 15 min. Next, the obtained white muscles, composed of 18.2% total protein, 2.4% lipids, and 1.3% ash, were chopped in a crusher (MQ 5025 plus, Braun GmbH, Frankfurt, Germany) for 15 s and blended with 2.5% (*w/w*) NaCl. According to the control group, labeled as SMSGs, the paste was chopped at 3000 rpm for 1 min with the moisture content adjusted to approximately 80% (*w/w*) through scrubbing the surface moisture from the minced surimi. For the experimental groups, KC, IC, and GG (1 g/100 g surimi) were incorporated into the aforementioned surimi and the mixture was further minced for 1 min. The samples were then stuffed into 50 mL centrifuge tubes and heated in two phases (45 °C for 30 min, 90 °C for 20 min). Finally, the samples were quickly cooled in an ice bath at approximately 4 °C for 30 min and then maintained at 4 °C overnight. During the entire preparation process, the samples were maintained at their natural pH without adjustment.

### 2.3. Gel Strength

Samples were set into barrels of 20 mm width and 10 mm height. Gel strength was tested via a TA-XT Plus texture analyzer (Stable Micro System, Surrey, UK) with a P/0.5 probe with the test speed of 1.0 mm/s, the compression variable of 50%, and the trigger force of 5.0× *g*, respectively. The measurement was performed with six replicates.

### 2.4. Texture Profile Analysis (TPA)

Samples were set into 20 mm width and 10 mm length cylinders. The hardness and chewiness of SMSG-based gels were analyzed via a TA-XT Plus texture analyzer (Stable Micro System, Surrey, UK) with a P/50 probe with test speed of 1.0 mm/s. The compression ratio and trigger force were set at 50% and 5.0× *g*, respectively. The test was performed with six replicates for each sample.

### 2.5. Sensory Evaluation

Three anionic polysaccharides (KC, IC, and GG) were solved in water at the same concentrations as those in the surimi system for sensory analyses. The sensory attributes were evaluated by a panel of 10 trained persons and classified into three grades (extremely sweet, slightly sweet, and odorless).

### 2.6. Water Holding Capacity (WHC)

Samples (1 g) were weighed (W_1_), placed in double-layer filter paper, and then transferred to 50 mL centrifuge tubes. After centrifugation at 5000× *g* for 20 min at 4 °C, the gels were rapidly taken out from the 50 mL centrifuge tubes and weighed again (W_2_). The WHC was measured via the equation below:

WHC (%) = (W_2_/W_1_) × 100

### 2.7. Low-Field Nuclear Magnetic Resonance (LF-NMR) Relaxometry

The MesoMR23-060V-1 analyzer (Niumag Analytical Instrument Co., Ltd., Shanghai, China) was utilized to record spin–spin relaxation time (*T*_2_) of SMSG-based gels at 32 °C with a 22.4 MHz resonance frequency and a 0.5 T magnetic field strength. The relevant parameters were determined as follows: τ = 100, *T*_W_ = 2500 ms, NECH = 5000, and NS = 8. The recorded *T*_2_ data were obtained via Multiexp Inv analysis software v1.06 according to the exponential distribution of Carr–Purcell–Meiboom–Gill decay curves.

### 2.8. Magnetic Resonance Imaging (MRI)

In accordance with Yan et al. [22] with some changes, *T*_1_ and *T*_2_ proton density images of SMSG-based gels were provided via spin-echo imaging sequence inside a 100 × 100 mm viewed field. The related parameters were set as follows: offset slice = 28.9 mm, read size = 256, and phase size = 192. The samples were set into three layers with a 2.0 mm width, and a 1.0 mm gap. The images were taken via a repetition time *T*_R_ = 500 ms and an echo time *T*_E_ = 20 ms for *T*_1_, and a repetition time *T*_R_ = 1600 ms and an echo time *T*_E_ = 50 ms for *T*_2_, respectively.

### 2.9. Dynamic Rheology Behavior

Samples were tested by a Discovery HR-1 rheometer (TA Instruments Menu Co., Ltd., New Castle, DE, USA) combined with a 40 mm parallel plate geometry. Surimi paste was scanned at a 1 Hz frequency and a 1 mm gap upon heating from 25 to 90 °C with the speed of 2 °C/min at 0.8% constant strain in agreement with the linear viscoelastic region via the oscillation amplitude mode.

### 2.10. Cryo-Scanning Electron Microscopy (Cryo-SEM)

In accordance with Yan et al. [23] and Zhang et al. [24] with some changes, the microstructures of SMSG-based gels were observed by a SU8000 SEM device (Hitachi Co., Ltd., Tokyo, Japan). The specimen was set in a holder and quickly immersed in liquid nitrogen slurry to be frozen. Next, the frozen specimen was directly placed under vacuum into the cryo-preparation chamber, and sequentially subjected to fracturing, sublimation (−60 °C for 40 min), and platinum sputtering (10 mV for 60 s). Finally, the prepared samples were transferred to a SEM chamber for imaging and recording via a 10.0 kV accelerating voltage at 5000× *g* magnification.

The acquired cryo-SEM images were processed via AngioTool64 software v0.5 (National Institute of Health, Bethesda, MD, USA) to calculate the quantitative parameters associated with quantitative network characteristics.

### 2.11. Raman Spectroscopy

The Raman spectra of samples were collected by a Raman spectrometer (LabRAM HR Evolution, Horiba Ltd., Kyoto, Japan) within the range of 400–3600 cm^−1^ by a 532 nm laser and 600 grating. The resolution was 1 cm^−1^ and the integration time was 30 s. The absorbance spectra were separated between 1600 and 1700 cm^−1^ to calculate the details of secondary structures via PeakFit software v4.12 (SPSS Inc., Chicago, IL, USA).

### 2.12. FTIR Spectroscopy

In accordance with Yan et al. [25] with some changes, lyophilized samples (1 mg) were wrapped in KBr (100 mg) followed by compression into a wafer before testing. The spectra were measured within a 4000–400 cm^−1^ scope, a 10 kHz scanning speed, a 4 cm^−1^ resolution of and 32 total scanning times.

### 2.13. Chemical Forces

According to Gu et al. [26] with some changes, the samples (0.5 g) were put into 20 mL SA solution (0.6 mol/L NaCl), homogenized at 6000 rpm for 2 min, agitated at 180 rpm for 1 h, and centrifuged at 10,000× *g* for 20 min. The supernatant was collected, and the sediment was homogenized with 20 mL SB solution (1.5 mol/L urea and 0.6 mol/L NaCl) by equal procedure. After that, the obtained sediment was subsequently redissolved in 20 mL SC solution (8 mol/L urea and 0.6 mol/L NaCl) twice. Finally, the achieved precipitation was redissolved in 20 mL SD solution (8 mol/L urea, 0.6 mol/L NaCl, and 0.5 mol/L β-mercaptoethanol). The quantity of ionic bonds, hydrogen bonds, hydrophobic interactions, and disulfide bonds was obtained by testing the protein amount in supernatants derived from SA, SB, SC, and SD solutions, respectively. The protein solubility (mg/g) was measured according to the ratio of dissolved protein content to total protein amount.

### 2.14. SDS-PAGE

The lyophilized samples were added into SDS-PAGE sample buffer (2×) at 2 mg/mL, heated at 100 °C for 5 min, and shaken continuously overnight at 25 °C to ensure adequate dissolution. The samples (8 μL) were set into the polyacrylamide gel. Finally, the gels were stained with Coomassie Brilliant Blue R-250 and destained by ethanol and glacial acetic acid. The band intensity was analyzed by ImageJ software v1.8.0 (National Institute of Health, Bethesda, MD, USA).

### 2.15. Statistical Analysis

The textural indices were examined a minimum of six times, while other tests were repeated in triplicate to ensure randomization and reproducibility for analysis. All recorded data were presented as mean ± standard deviation. One-way ANOVA followed by Duncan’s multiple range test was applied for significantly different means by SPSS 25.0 software (SPSS, Inc., Chicago, IL, USA). A level of *p* < 0.05 was determined as significant.

## 3. Results and Discussion

### 3.1. Effect of Polysaccharides on Gel Strength of SMSGs

**Figure 1:**
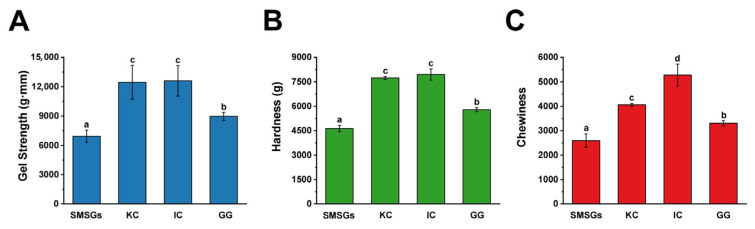
Gel properties of SMSGs modified by KC, IC, and GG. (**A**) Gel strength. (**B**) Hardness. (**C**) Chewiness. Different letters (a–d) suggest significant differences (*p* < 0.05) among the groups.

As reported, the concentrations of KC, IC, and GG used to modify surimi gels are in the range of 0.04–2%, 0.25–1%, and 0.05–8%, respectively [13,14,15,16,27]. Therefore, we selected the common concentration at 1% to study the comparison effects of KC, IC, and GG on SMSG properties. The gel strength of SMSGs with KC, IC, and GG treatments is presented in Figure 1A. In comparison to SMSGs, the incorporation of KC, IC, and GG significantly increased gel strength (*p* < 0.05), as reflected by 79.4%, 81.5%, and 29.3% increments, respectively, in which carrageenan had more significant improvement effects than GG. To some extent, carrageenan is a strongly anionic polysaccharide with high charge density [28], resulting in superior gel strength behavior in SMSGs.

Typically, myofibrillar proteins are the major constituents associated with the gelling process in raw surimi [13]. Research by Chen et al. [1] has stated that the intervention of 0.1–0.4% curdlan, KC, or gelatin could apparently promote the gel strength of silver carp surimi with increments of 8.7–50.2%, which might be due to the expansion and water absorption of polysaccharides during heating, thus filling the network structure of myofibrillar proteins in the surimi gels by a cooling procedure. As reported, GG is more likely to form polymers and aggregate with surimi proteins during setting or heating, thereby strengthening the gel matrix [16]. In addition, carrageenan is structurally a linear, sulfated anionic polysaccharide, which could prompt the gelation of myofibrillar proteins in surimi [29]. Thus, it is assumed that the gel strength of SMSGs could be enhanced by adding KC, IC, and GG, which might be mainly due to the existence of sulfate groups in carrageenan and the carboxyl groups in GG.

### 3.2. Effect of Polysaccharides on TPA of SMSGs

TPA was applied to simulate the chewing of foods twice to obtain the characteristic values of the gel matrix, which could indicate the flavor of surimi products and the acceptability and preference of customers [30]. TPA indices including hardness and chewiness were chosen as representative parameters to evaluate the texture properties of SMSGs modified by KC, IC, and GG (Figure 1B,C). The hardness of samples is represented by the peak force of the force–time curve in the first compression cycle [31]. A significant increment in hardness was observed when adding KC, IC, and GG (*p* < 0.05), with increases of 71.3%, 66.9%, and 24.9%, respectively, in comparison to that in SMSGs (Figure 1B), indicating favorable compatibility between polysaccharides and surimi proteins. Chewiness measures the energy needed to masticate foods to the point where they can be swallowed [1]. As depicted in Figure 1C, the chewiness of the KC-, IC-, and GG-treated groups was significantly greater than that of SMSGs with increases of 56.5%, 103.4%, and 27.4% (*p* < 0.05), respectively, being the same as the hardness results (Figure 1B). In this case, the variation trend of TPA results was consistent with that of the gel strength results in the SMSG system. Typically, gel strength and TPA are two essential indices utilized to reflect the structural integrity and sensory attributes of the textural properties in gel samples [32]. Researchers have also found that the TPA results show a positive correlation with gel strength results in Pacific cod surimi/soy protein isolate/KC [32], bigeye snapper surimi/GG [16], and silver carp surimi/sulfated polysaccharide [33] systems, fully investigating the texture performance of gel matrices in terms of both structural integrity and sensory properties.

Collectively, Petcharat and Benjakul [16] found that the enhanced texture behavior of bigeye snapper surimi modified by GG at 2–8% showed a 2.4–38.0% increase, which might be associated with the binder or filler effect of GG and the increase in hydration among the protein matrix and GG. Additionally, the sulfate groups existing in KC and IC might promote protein cross-linking in surimi [20]. Li et al. [34] have also proposed that KC could induce the unfolding of myofibrillar proteins in Antarctic krill, leading to spatial conformation alterations and side-chain group exposure within the gel system. Therefore, it is suggested that KC, IC, and GG could improve the textural properties of SMSGs due to the enhanced interactions between functional groups of polysaccharides and myofibrillar proteins. In addition, we further explored the sensory properties of SMSGs modified by KC, IC, and GG. We evaluated the taste of KC, IC, and GG aqueous solutions at the same concentrations as those in the surimi system. To be specific, KC was odorless, and IC as well as GG presented a slightly sweet taste. Consequently, we confirmed that KC, IC, and GG did not affect the sensory acceptability of SMSGs. Therefore, it is suggested that these three anionic polysaccharides could be desirable exogenous additives for SMSG, reflected by the fact that they maintained its sensory attributes in addition to improving its gel properties.

### 3.3. Effect of Polysaccharides on WHC of SMSGs

**Figure 2:**
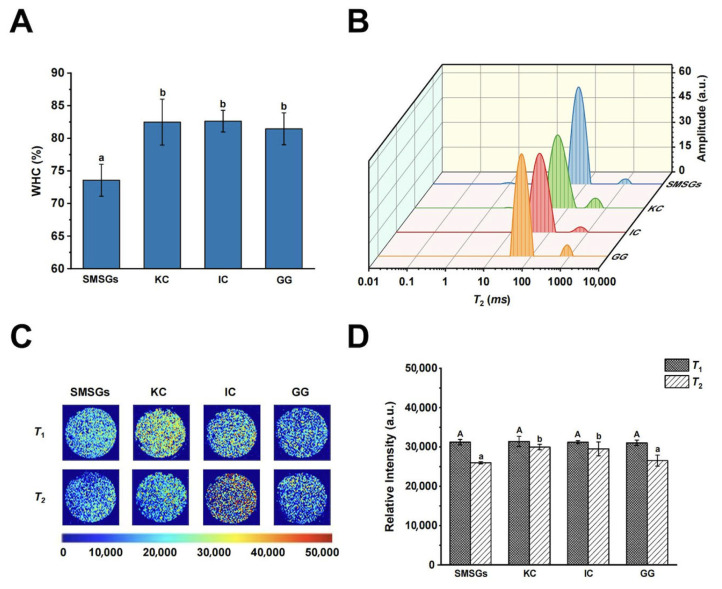
Water status of SMSGs modified by KC, IC, and GG. (**A**) WHC. (**B**) *T*_2_ relaxation time profiles. (**C**) *T*_1_ and *T*_2_ weight images. (**D**) Relative intensity of *T*_1_ and *T*_2_ weight images. The capital letter (A) was utilized to imply the significant difference in *T*_1_ (*p* < 0.05) among the groups, and the small letters (a–b) were utilized to imply the significant difference in WHC and *T*_2_ (*p* < 0.05) among the groups.

WHC is a quantitative index to reflect the water retention ability of surimi gels [1]. The WHC changes in SMSGs by adding KC, IC, and GG are shown in Figure 2A. The WHC values of SMSGs containing KC, IC, and GG were significantly increased by 13.1%, 12.1%, and 10.7% compared with that of SMSGs alone, respectively (*p* < 0.05). These results could further confirm the electronic interactions between the negatively charged domains of sulfate groups in KC and IC, the carboxyl group in GG, and the positive residues of myofibrillar proteins. Lan et al. [14] have reported that the inclusion of 0.04–0.22% KC could absorb water and expand, thus putting pressure on the gel structure of golden pompano (*Trachinotus blochii*) surimi belonging to the family of pompanos, reflected by a significant increase of approximately 4.6–12.3% in WHC (*p* < 0.05), which could bind more water and water-soluble substances. Typically, a gelling network is formed by combining moisture or trapping other ingredients during the heat-induced gelation process [30]. The improved WHC could also be related to the great combination between the hydrophilic groups of water-soluble proteins in surimi and water molecules, thus promoting the formation of molecular bonds such as hydrogen bonding to capture enough water within gel networks [13]. Therefore, it is assumed that KC, IC, and GG facilitate water locking in the gel matrix, thereby enhancing the WHC of mixed gel systems.

### 3.4. Effect of Polysaccharides on Water Migration and Distribution of SMSGs

LF-NMR and MRI are useful methods exhibiting great potential for non-destructive measurements and quantitative visualization of water migration and distribution in food systems [35,36]. According to the LF-NMR data, the *T*_2_ relaxation time of the SMSGs containing KC, IC, and GG is shown in Figure 2B. Three peaks of the *T*_2_ curve were detected in the samples, with *T*_2_ in the range of 1–10 ms (*T*_21_), 30–200 ms (*T*_22_) and 200–1000 ms (*T*_23_), representing bound water, immobile water, and free water, respectively [12], except for the IC- and GG-treated groups with only two peaks (*T*_22_ and *T*_23_), implying the alteration of water status within the gel matrices. Moreover, Zhang et al. [37] also observed an LF-NMR curve with different amounts of peaks in a crocodile myofibrillar protein/KC gel system. In particular, immobile water was the primary composition of water filling in the gel network, with the percentage exceeding 90% of the total signals. The results showed that the *T*_21_ peak showed the lowest value with no significant change, while both *T*_22_ and *T*_23_ were significantly shortened by adding polysaccharides to surimi gels with decreases from 61.34 to 52.43–55.93 ms and 1106.95 to 503.19–970.60 ms, respectively (*p* < 0.05) (Table 1). These observations revealed the promotion of the combination of SMSGs and water by adding polysaccharides, leading to reduced water mobility being related to the microstructure and matrix rigidity of surimi products. Yu et al. [38] and Lan et al. [14] have also reported that the *T*_22_ and *T*_23_ values of sea cucumber compound surimi gels modified by 0.1% KC and golden pompano surimi gels with 1% KC added shifted to lower relaxation times, with an obvious blueshift from 75.79 to 64.46 ms and 49.97 to 44.71 ms in *T*_22_ as well as a significant blueshift from 821.43 to 580.52 ms and 460.84 to 368.67 ms in *T*_23_ compared with those of the control group, respectively (*p* < 0.05), suggesting that KC could possibly restrict water movement via hydrogen and hydrophobic bonds formed by the anionic sulfate groups in KC and water molecules, thus facilitating the formation and cross-linking of a gel network of surimi gels. Thus, it is suggested that polysaccharides could reduce the movement of water molecules through strengthening the link within the gel network and water molecules.

For the water migration analysis, redder and brighter proton images indicate higher hydrogen proton density in surimi gels, while bluer or darker images represent lower hydrogen proton signal intensity [22]. As depicted in Figure 2C, *T*_1_ showed significantly greater signal densities than that in *T*_2_, suggesting that water with a lower relaxation time was prevalent in SMSG-based samples, which was consistent with the LF-NMR results (Figure 2B). For *T*_1_ weight images, the signal density exhibited minor changes among all groups (*p* > 0.05). For *T*_2_ weight images, the signal density displayed a significant increase by adding KC and IC (*p* < 0.05), while the GG-treated group exhibited no significant change (*p* > 0.05) (Figure 2D). As reported, curdlan and tamarind seed polysaccharide have been added into *Nemipterus virgatus* and grass carp surimi gels, leading to an increased red color area of the proton NMR images, indicating the water migration restriction, uniform water distribution, and great WHC of the surimi gels in the presence of polysaccharides [39,40]. Therefore, it is suggested that incorporating KC and IC into SMSGs could effectively enhance the binding capability of SMSGs with water, resulting in more hydrogen protons within the network.

### 3.5. Effect of Polysaccharides on Dynamic Rheology of SMSGs

**Figure 3:**
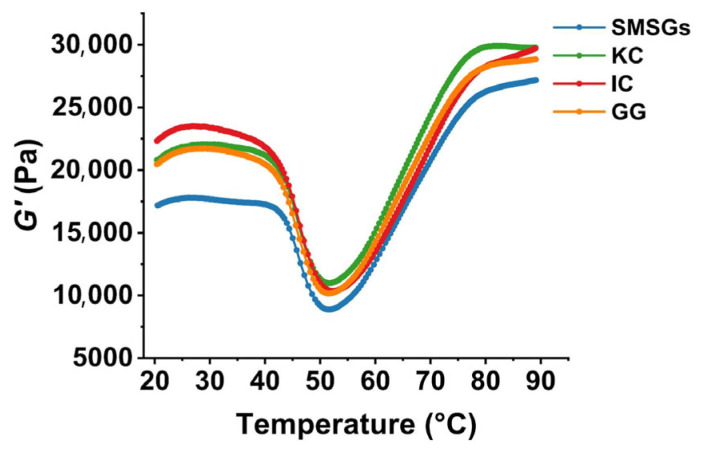
The thermal stability characteristics of SMSGs modified by KC, IC, and GG.

The changes in viscoelasticity during the process from sol to gel of surimi samples can be detected by dynamic rheology [32]. The storage modulus (*G*′) is a vital index to describe the unfolding and aggregation processes of surimi proteins during the heating process [35]. As shown in Figure 3, SMSG-based samples exhibited similar gelation patterns characterized by three stages of gel formation, gel weakening, and gel enhancement. During the initial heating phase, *G*′ increased slowly and peaked at around 30 °C, mainly due to the aggregation of partially unfolded actomyosin, thus forming a preliminary gel network via hydrogen bonds [41]. Afterwards, *G*′ began to decrease with the increasing temperature until heating to around 52 °C, attributing to the heating breaking of hydrogen bonds and myosin degradation resulting from the heat-activated endogenous proteases [42]. As the temperature continued to rise to 90 °C, the *G*′ exhibited a further elevation, indicating the formation of a stable gel structure [43]. After adding polysaccharides, *G*′ was significantly higher than that in SMSGs during the whole heating stage (Figure 3). In detail, the increasing folds of the KC-, IC-, and GG-treated groups were 1.09, 1.09, and 1.06 in comparison to SMSGs at 90 °C, respectively, suggesting the promotion effect of polysaccharides on strengthening network structure formation during the gelation process. Similarly, Zhang et al. [40] and Zhu et al. [2] have studied surimi gelation improvement by curdlan and konjac glucomannan treatments, where *G’* was significantly higher after polysaccharide addition, indicating the enhancement of the thermal stability of surimi proteins modified by curdlan and konjac glucomannan. Therefore, it is suggested that KC, IC, and GG could promote the suitable denaturation and aggregation of surimi proteins, leading to the enhancement of the thermal stability and elasticity of SMSG-based samples.

### 3.6. Effect of Polysaccharides on Cryo-SEM of SMSGs

**Figure 4:**
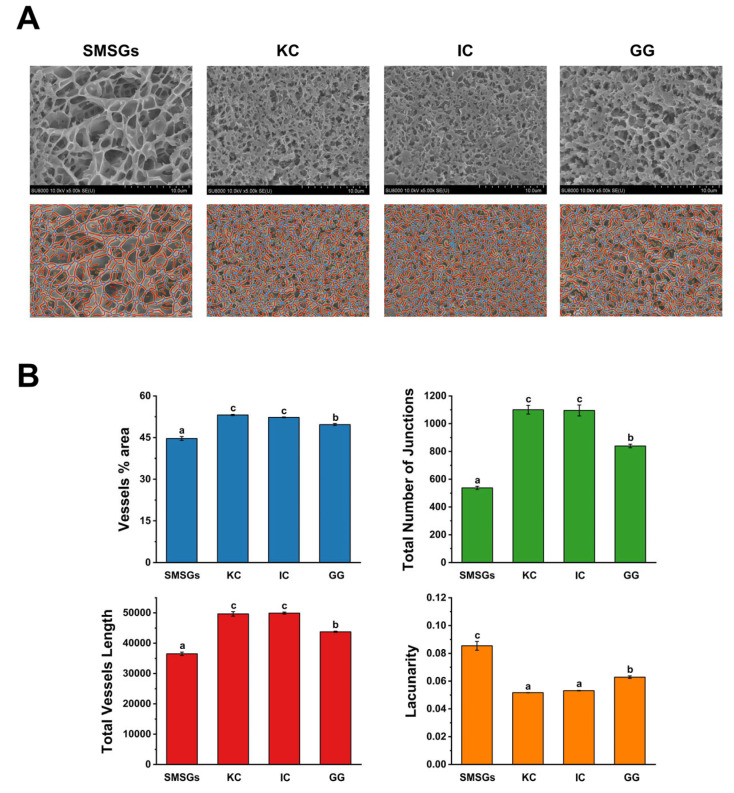
Microstructures of SMSGs modified by KC, IC, and GG. (**A**) Cryo-SEM images. (**B**) Quantitative network analysis parameters including vessel area (yellow lines), total number of junctions (blue dots), total vessel length (red lines), and lacunarity (empty areas). Different letters (a–c) suggest significant differences (*p* < 0.05) among the groups.

The gel performance of surimi gels is closely related to their internal microstructures, and could be detected by cryo-SEM, which could maintain the authentic state of the samples maximally at a submicron level [44]. As described in Figure 4A, the SMSGs formed a coarse and disordered gel network with sizable nonuniform holes related to the impaired interaction of myofibrillar proteins, leading to lower gel strength (Figure 1A). The KC-, IC-, and GG-treated groups exhibited the notable improvement on the compactness of the gel network with the distribution of dense and homogeneous pores. These results could certainly explain the great gel properties of SMSGs modified by KC, IC, and GG (Figure 4A), where a contracted and dense gel network generally contributes to improved gel quality [2]. A study by Petcharat and Benjakul [16] found that adding 6% GG could cause the bigeye snapper surimi gel network to become denser, ascribing to the ionic interaction within negative charge of carboxyl groups in GG and positive patches of myofibrillar proteins. A study by Huang et al. [35] has noted that the composited surimi gels composed of inulin and KC showed a compact and well-aggregated framework, possibly due to the stabilization of the moisture content and reduction in water channel formation within the gel network caused by inulin and KC.

Correspondingly, the quantized indices, such as vessel percentage area, total number of junctions, total vessel length, and lacunarity, associated with network features of SMSGs modified by KC, IC, and GG are shown in Figure 4B. The variations in these quantized parameters could effectively reflect gel networks, such as the structural area ratio, pore size, and continuity [45]. The control sample exhibited relatively loose networks with large voids, exhibiting the lowest vessel percentage area, total number of junctions, and total vessel length, and the highest lacunarity. By intervening with KC, IC, and GG, the vessel percentage area, total number of junctions, and total vessel length of SMSGs significantly increased by 11.1–18.9%, 56.2–104.8%, and 19.8–36.6%, respectively, while lacunarity significantly decreased by 26.3–39.5% (*p* < 0.05), corresponding to massive network wall segments with a more compact porous connected gel network, as shown in Figure 4A. Indeed, structure pore size is closely correlated with lacunarity. In detail, networks with homogenous and compact pores exhibit comparatively lower lacunarity, while those with odd gaps and voids conversely display higher lacunarity [23]. Accordingly, the vessel area was labeled with yellow lines, the total number of junctions was enclosed with blue dots, total vessel length was marked with red lines, and lacunarity reflected the degree of empty areas (gray areas free from yellow, red, and blue colors) within the network. Moreover, the water status of surimi gels reflected by the variation trends of WHC and *T*_2_ relaxation times are closely related to the pore size of the gel network structure [39]. To be specific, the KC-, IC-, and GG-treated groups promoted left shifts in *T*_22_ to the lower values (Figure 2B), leading to more water retention within the mixed surimi gel structure with finely distributed pore sizes, which correspond to the results of cryo-SEM and relevant quantitative network analysis (Figure 4A,B). Thus, it is assumed that the homogeneous gel network with a high vessel area, total number of junctions, and total vessel length as well as a low lacunarity could be observed in SMSGs containing KC, IC, and GG, leading to more water being entrapped in the gel matrix as reflected by enhanced WHC and texture attributes.

### 3.7. Effect of Polysaccharides on Secondary Structure of SMSGs

**Figure 5:**
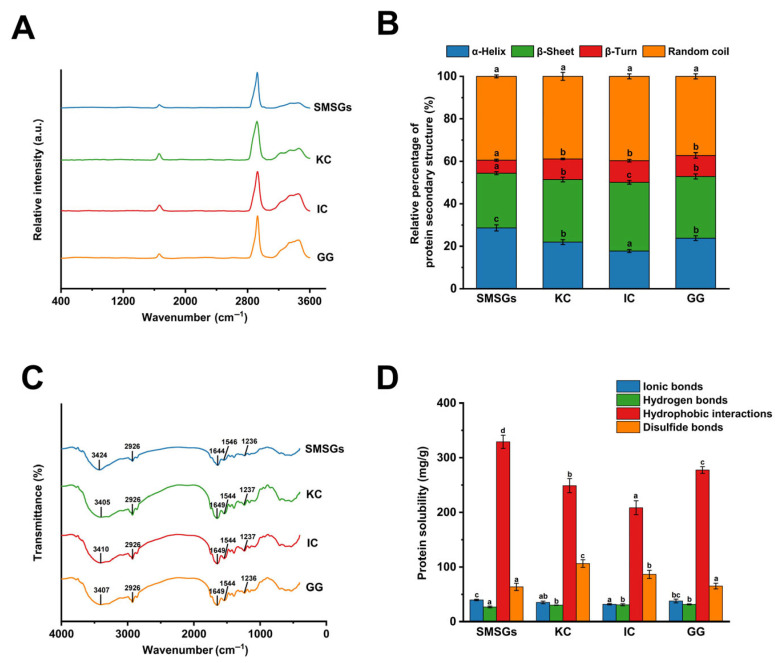
Conformational properties and molecular interactions of SMSGs modified by KC, IC, and GG. (**A**) Raman spectra. (**B**) Protein secondary structure. (**C**) FTIR spectra. (**D**) Chemical forces. Different letters (a–d) suggest significant differences (*p* < 0.05) among the groups.

Raman spectroscopy was implemented to analyze the conformation of SMSGs modified by polysaccharides (Figure 5A). The typical peaks of amide I (primarily C=O stretching) and amide III (mainly in-plane N-H bending and C-N stretching) bands are vital for evaluating protein secondary structure [46]. Specifically, the bonds centered around 1650–1658, 1665–1680, 1680–1690, and 1660–1665 cm^−1^ are representative of α-helix, β-sheet, β-turn, and random coil, respectively [47]. As depicted in Figure 5B, the KC, IC, and GG incorporation facilitated a significant decrease in the relative proportion of α-helix accompanied by a significant increment of the relative percentage of β-sheet and β-turn in SMSGs (*p* < 0.05), indicating conformational changes from α-helix to others. Similarly, Chen et al. [1] and Mi et al. [12] have reported a diminution of the proportion of α-helix along with an enhancement of the content of β-sheet in silver carp surimi gels modified by curdlan and KC, revealing the unfolding of α-helix and the production of β-sheet, thus promoting generation of a more homogeneous gel network. Indeed, a higher content of β-sheet structure evinces an improvement of intermolecular hydrogen bonding within peptide chains and an ordered reorganization of protein molecules, resulting in greater gel properties and a more ordered network structure [48]. Thus, it is assumed that KC, IC, and GG raise the percentage of β-sheet and β-turn at the expense of α-helix fractions, indicating better gel performance and a more ordered gel network.

### 3.8. Effect of Polysaccharides on FTIR Spectra of SMSGs

FTIR spectra were acquired to investigate the alterations in the functional groups of components, thereby indicating the changes in intermolecular or intramolecular interactions between different ingredients [49]. As presented in Figure 5C, SMSGs exhibited a broad peak at 3424 cm^−1^ generated via the tensile stretching of O-H groups. Compared with the hydroxyl groups of SMSGs, the absorption peak revealed apparent blue shifts to 3405, 3410, and 3407 cm^−1^ in the spectra of the KC-, IC-, and GG-treated groups, respectively, indicating stronger hydrogen bond formation between SMSGs and polysaccharides. Furthermore, three characteristic peaks were observed in SMSGs at 1644, 1546, and 1236 cm^−1^, corresponding to the amide I, II, and III bands, respectively. In particular, the amide I bands of SMSGs containing KC, IC, and GG all showed an obvious red shift to 1649 cm^−1^, symbolizing electrostatic interactions existing in SMSG-based samples. To some extent, amide I is the peak attributed to C=O stretching, where amide I band movement could reflect the protein secondary structure changes in SMSGs-based samples confirmed by the Roman spectra results (Figure 5A,B). In total, the SMSGs after adding polysaccharides displayed similar FTIR patterns to the single SMSGs without introducing any new bands, indicating that SMSGs did not produce new functional groups after the addition of polysaccharides. Li et al. [34] obtained similar results by evaluating the improvement effect of KC on the gel qualities of Antarctic krill myofibrillar proteins, where blueshifts were observed in amide A bands from 3414 cm^−1^ to 3292 cm^−1^, revealing that a synergistic effect occurred within myofibrillar proteins and KC via strengthened hydrogen bonding. Moreover, the typical peaks including the O–H bands, amide I, and amide II groups of threadfin bream surimi gels also show a blue or red shift after adding psyllium husk powders at 1–4% within the ranges of 3322–3276, 1642–1634, and 1538–1535 cm^−1^, respectively [50]. These vibrations indicate the interactions, mostly via H-bonding, between functional groups of surimi proteins and polysaccharides of psyllium husk powders, which might be related to the dilution of proteins by psyllium husk powder addition. Therefore, these results reveal the changes in protein secondary structure and the involvement of hydrogen bonding and electrostatic interactions of SMSGs after the addition of KC, IC, and GG.

### 3.9. Effect of Polysaccharides on Chemical Forces of SMSGs

The stability of the protein conformation is primarily supported by chemical forces, and their compositions determine the gel properties of surimi samples [26]. As depicted in Figure 5D, SMSG-based samples showed relatively higher value in hydrophobic interactions and disulfide bonding, followed by ionic bonds and hydrogen bonding, suggesting that hydrophobic forces and disulfide bonds were major forces affecting construction and stability of surimi gels. Similar phenomena have been observed in Antarctic krill and hairtail surimi gel systems [20,51]. In addition, the ionic bonds were significantly decreased by adding KC and IC compared with that of SMSGs, while the hydrogen bonds exhibited a significant upward trend with the addition of polysaccharides (*p* < 0.05) (Figure 5D). Moreover, the variation trends of ionic and hydrogen bonds of SMSG-based samples were identical to our FTIR results (Figure 5C). These observations revealed the promotion of the cross-linking of SMSGs caused by polysaccharide involvement. Indeed, the ionic bonds existing within the SMSG system could verify ionic interactions between the negatively charged residues of polysaccharides and myofibrillar proteins in SMSGs. Generally, hydrophobic interaction is also an important index for protein gelation, where the myofibrillar protein tail molecules unfold, resulting in the change in protein spatial conformation and hydrophobic amino acid exposure to the water environment during the surimi heating process [52]. As presented in Figure 5D, the hydrophobic interaction was the main chemical bond in SMSG-based samples, revealing the great hydrophobicity and stability of the gel system and the activation of hydrophobic groups in myofibrillar proteins after introducing polysaccharides. Moreover, the high amount of disulfide bonds in surimi gels might be caused by the unfolding of sulfhydryl groups under heating [13]. In comparison to those of SMSGs, the disulfide bonds of SMSGs modified by KC and IC were significantly enhanced (*p* < 0.05) (Figure 5D), revealing the exposure of more sulfhydryl groups for oxidation. Yan et al. [13] found similar results for improving disulfide bonds in minced scallop gels modified by curdlan and KC, inferring that aggregation of the myofibrillar protein head molecules forms disulfide bonds. Therefore, it is suggested that SMSGs containing various polysaccharides are primarily maintained by hydrophobic interactions and disulfide bonds, and myofibrillar protein head molecule aggregation in SMSGs could be increased by KC and IC incorporation.

### 3.10. Effect of Polysaccharides on SDS-PAGE of SMSGs

**Figure 6:**
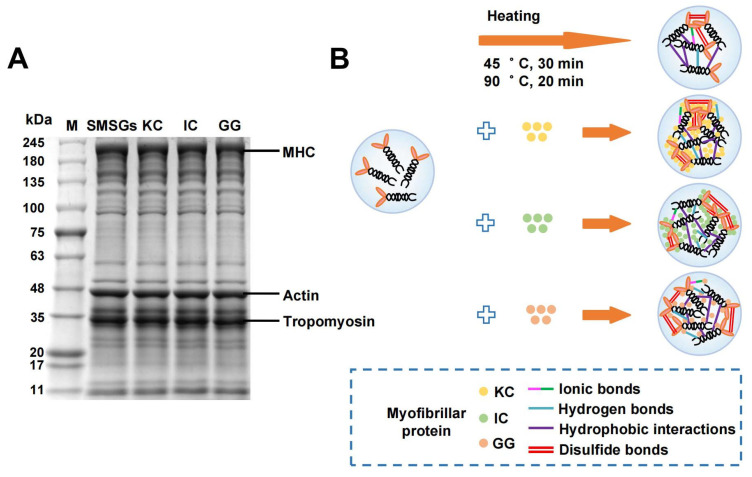
(**A**) SDS-PAGE and (**B**) mechanism diagram of SMSGs modified by KC, IC, and GG.

The influence of polysaccharides on electrophoretic patterns of the surimi gels was depicted in Figure 6A. For surimi samples, the major protein components were myosin heavy chain (MHC), actin, and tropomyosin according to the bond intensity measured by Image J software v1.8.0. Clearly, there was no obvious change in the intensity of primary protein bands in each sample, indicating that the participation of polysaccharides had no significant influence on the polymerization or protein degradation in SMSGs. In detail, the bond intensity ratios of MHC, actin, and tropomyosin were in the ranges of 20.7–23.7%, 15.8–17.1%, and 25.2–27.1%, respectively (Table S1). Similar studies have found that adding a small amount of polysaccharides could not alter the strength or position of the characteristic band of surimi proteins, in which the polysaccharide might act as a functional filler within the surimi gel network [53]. Yu et al. [38] have also noted that there is no chemical alteration in proteins modified by KC, probably because of the electrostatic bridging effects of KC. Therefore, it is suggested that the major protein bonds of SMSGs show no obvious change with the addition of various polysaccharides, suggesting no protein composition alteration concurring with polysaccharide intervention.

### 3.11. The Formation Mechanism of SMSGs with the Addition of Various Polysaccharides

The proposed reaction mechanism of SMSGs modified by various polysaccharides is shown in Figure 6B. The myofibrillar proteins in SMSGs tend to aggregate by head-to-head and tail-to-tail binding after heating. Based on the results mentioned above, the involvement of KC, IC, and GG could facilitate the compact gel network formation of SMSGs due to the filling effect of polysaccharides. In detail, the intermolecular forces of SMSG-based gels were mainly governed by the hydrophobic interactions within the myofibrillar protein tails and disulfide bonds within the myofibrillar protein heads. The improvement effect of KC and IC on the gel properties of SMSGs could be mainly attributed to the increased content of disulfide bonds, expressed as the sufficient cross-linking and aggregation of myofibrillar protein head molecules in SMSGs. Additionally, GG caused strengthened hydrogen bonds within the abundant hydroxyl groups in GG and myofibrillar protein molecules in SMSGs, thereby improving the gel properties of SMSGs. Therefore, it could be inferred that polysaccharides effectively facilitate the disulfide bonds and hydrogen bonds of myofibrillar proteins in SMSGs, thus leading to superior gelation capacity.

## 4. Conclusions

The gel properties of SMSGs were modified by adding three anionic polysaccharides. To be specific, KC, IC, and GG noticeably enhanced gel strength, hardness, and chewiness and increased WHC by binding more water entanglement within the structure, thus resulting in a more compact and uniform gel network. Moreover, the two forms of carrageenan had more improvement effects than GG. Furthermore, the involvement of polysaccharides facilitated the structural conversion of SMSG proteins from α-helix to β-sheet and β-turn. The thermal stability could be improved by adding polysaccharides, probably due to the interactions between polysaccharides and surimi proteins, which were mainly composed of hydrophobic interactions and disulfide bonds. The present study focused on comparing the effects of three anionic polysaccharides on the physical characterization of SMSGs. To clarity the synergistic effect of carrageenan on the quality properties of SMSGs, we plan to conduct a further study into the interaction mechanisms between carrageenan and myofibrillar protein in Spanish mackerel.

## Figures and Tables

**Table 1:** Relaxation times of SMSGs modified by KC, IC, and GG.

Samples	*T*_21_ (ms)	*T*_22_ (ms)	*T*_23_ (ms)
SMSGs	1.74 ± 0.80 ^a^	61.34 ± 1.70 ^c^	1106.95 ± 15.32 ^c^
KC	3.02 ± 0.45 ^b^	52.43 ± 1.66 ^a^	503.19 ± 23.79 ^a^
IC	-	55.93 ± 1.80 ^b^	752.93 ± 171.35 ^b^
GG	-	55.92 ± 0.89 ^b^	970.60 ± 0.80 ^bc^

Data were expressed as means ± SD from triplicate determinations. Different letters in the same column suggest significant differences (*p* < 0.05).

## Data Availability

The original contributions presented in the study are included in the article/Supplementary Materials. further inquiries can be directed to the corresponding authors.

## Supplementary Materials

The following supporting information can be downloaded at: https://www.mdpi.com/article/10.3390/foods14152671/s1, Table S1: Myosin heavy chain, action, and tropomyosin bands intensity proportion of SMSGs modified by KC, IC, and GG.

## Abbreviations

SMSGs	Spanish mackerel surimi gels
KC	κ-carrageenan
IC	ι-carrageenan
GG	gellan gum
TPA	texture profile analysis
WHC	water-holding capacity
LF-NMR	low-field nuclear magnetic resonance
*T*_2_	Spin–spin relaxation time
MRI	magnetic resonance imaging
FTIR	Fourier transform infrared
Cryo-SEM	cryo-scanning electron microscopy
SDS-PAGE	sodium dodecyl sulfate–polyacrylamide gel electrophoresis
MHC	myosin heavy chain
