# Supplementary material for: Modification of Spanish Mackerel (Scomberomorus niphonius) Surimi Gels by Three Anionic Polysaccharides
Source: Foods. 2025 Jul 29;14(15):2671. doi: 10.3390/foods14152671 (PMC12346181; doi:10.3390/foods14152671)
Supplement: Supplementary file 1 [file foods-14-02671-s001.zip › foods-3725685-supplementary.pdf]

Table S1 Myosin heavy chain, action, and tropomyosin bands intensity proportion of SMSGs modified by KC, IC, and GG.

Sample	Band intensity proportion (%)		
	Myosin heavy chain	Action	Tropomyosin
SMSGs	23.7	17.1	27.1
KC	21.5	15.8	26.2
IC	20.7	15.9	25.2
GG	21.1	16.3	26.2

Data were expressed as means±SD from triplicate determinations. Different letters in the same column indicated significant differences ($P < 0.05$).
